# Experimental and Computational Study of a Liquid Crystalline Dimesogen Exhibiting Nematic, Twist-Bend Nematic, Intercalated Smectic, and Soft Crystalline Mesophases

**DOI:** 10.3390/molecules26030532

**Published:** 2021-01-20

**Authors:** Emily E. Pocock, Richard J. Mandle, John W. Goodby

**Affiliations:** 1 Department of Chemistry, University of York, Heslington, York YO10 5DD, UK; ep851@york.ac.uk; 2 School of Physics and Astronomy, University of Leeds, Leeds LS2 9JT, UK

**Keywords:** liquid crystal, nematics, X-ray scattering, N_TB_

## Abstract

Liquid crystalline dimers and dimesogens have attracted significant attention due to their tendency to exhibit twist-bend modulated nematic (N_TB_) phases. While the features that give rise to N_TB_ phase formation are now somewhat understood, a comparable structure–property relationship governing the formation of layered (smectic) phases from the N_TB_ phase is absent. In this present work, we find that by selecting mesogenic units with differing polarities and aspect ratios and selecting an appropriately bent central spacer we obtain a material that exhibits both N_TB_ and intercalated smectic phases. The higher temperature smectic phase is assigned as SmC_A_ based on its optical textures and X-ray scattering patterns. A detailed study of the lower temperature smectic ‘’X’’ phase by optical microscopy and SAXS/WAXS demonstrates this phase to be smectic, with an in-plane orthorhombic or monoclinic packing and long (>100 nm) out of plane correlation lengths. This phase, which has been observed in a handful of materials to date, is a soft-crystal phase with an anticlinic layer organisation. We suggest that mismatching the polarities, conjugation and aspect ratios of mesogenic units is a useful method for generating smectic forming dimesogens.

## 1. Introduction

Liquid crystals can be broadly defined as any state of matter with some degree of positional or orientational organisation intermediate between the isotropic liquid state and a crystalline solid with long-range positional and orientational order in three dimensions. For example, the nematic phase possesses only short-range orientational organisation whilst smectic phases also exhibit positional order in one dimension.

The experimental discovery of nematic polymorphism in the early 21st century (i.e., new nematic phase types) has provided fresh impetus to the study of nematic liquid crystals [1,2,3]. The most well-known example of nematic polymorphism is the twist-bend modulated nematic phase (N_TB_), which possesses a helical structure with a pitch length of a few nanometers [4,5,6] and is therefore chiral in spite of being typically formed by achiral molecules, although a handful of chiral materials are known to exhibit this phase [7,8,9]. The N_TB_ phase exhibits striking optical textures [10] and has been studied by resonant [4,11] and non-resonant SAXS, [12] NMR, [13,14,15] polarised Raman spectroscopy, [16] and under applied electric [17] and magnetic fields. [18] The twist-bend nematic phase is principally formed by liquid-crystalline dimers, [19] in which two rigid sections are adjoined by a (semi) flexible spacer. However, this phase of matter is also observed in liquid crystalline n-mers, [20,21] hydrogen bonded systems, [22,23] bent-core materials, [24] and non-linear oligomers. [25] Experimental results show the importance of molecular shape [26,27,28,29] and the gross bend-angle in dictating the occurrence of this phase, [28,30,31] largely supporting the findings of earlier theoretical treatments. [32,33] 

It is now largely trivial to design new materials that will exhibit nematic and N_TB_ mesophases, pairing an appropriate central spacer (dictates bend angle and conformer distribution) with suitable mesogenic units (dictates transition temperatures); this is reflected by the fact that hundreds of materials are now known to exhibit this phase [34,35,36,37,38].

Transitions from the N_TB_ phase into other phases are still rare, and just a few examples of such transitions to smectic [39,40,41,42,43,44] and B_6_ phases [24,45] are known. The behaviour of key physical properties across such phase transitions, for example, elastic constants, N_TB_ pitch length, helicoidal angle, etc., would be expected to give insight into the nature of the self-organising process that leads to the formation of the N_TB_ state. While the relationship between the N_TB_ phase and molecular structure is understood to a certain extent [28,36,39,46,47,48,49,50] there is no such relationship known for materials that exhibit both N_TB_ and smectic phases and, at least in the experience of the authors, these have been discovered on a largely serendipitous basis. Previously we reported that increasing the length of terminal alkyl chains is a useful way to generate smectic phases in dimers and bimesogens, [36] however this typically leads to diminished nematic and N_TB_ phase stability and so we sought to avoid this stratagem. 

The cyanobiphenyl unit commonly used in dimers tends to self-associate into antiparallel pairs through dipole-dipole interactions, [51] some fluorinated mesogenic units do not. [52,53] We considered that replacement of one cyanobiphenyl unit in a simple dimer (CB8OCB) [30,54] with a 3,4,5-trifluoroterphenyl unit (FFFT) might lead to segregation of the different units into alternate layers and thus promote the formation of smectic phases. The difference in the size of the two mesogenic units (CB ≈ 9.7 Å, FFFT ≈ 12.8 Å, at the DFT(B3LYP/6-31G(dp) level) might also be expected to lead to segregation and thus the formation of smectic phases directly from the N_TB_ phase. 

## 2. Results and Discussion

### 2.1. Mesomorphic Behaviour

The transition temperatures of **CB8OFFFT** were determined by polarised optical microscopy and differential scanning calorimetry (DSC) and are presented in Table 1.

**Figure 1:**
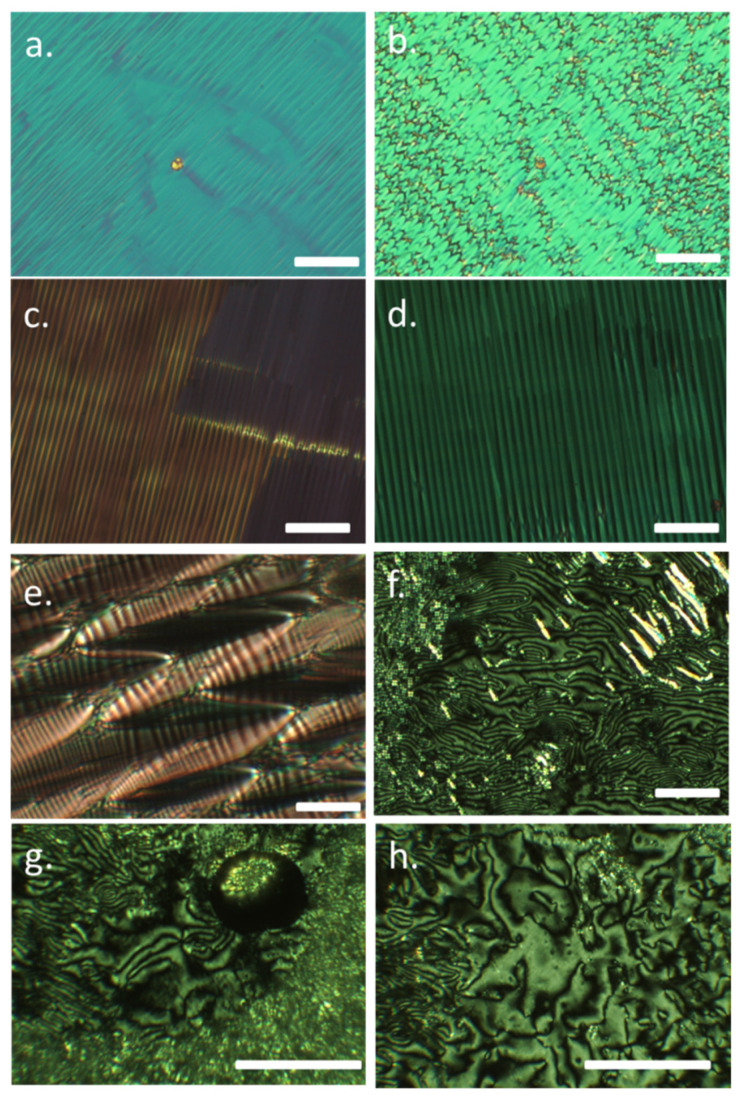
Photomicrographs of compound **CB8OFFFT**: (**a**) the rope texture of the twist-bend phase forming at 94.5 °C; (**b**) parabolic defects in the SmC_A_ phase at 92 °C; (**c**) slow cooling (0.1 °C min^−1^) across the nematic (N_TB_)-SmC_A_ transition shows the transition front, the rope texture of the N_TB_ (left) is retained in the SmC_A_ phase (right) with a significant change in birefringence (93.3 °C); (**d**) rope-texture of the SmC_A_ phase at 85 °C; (**e**) striations visible in the focal-conic texture of the SmC_A_ phase at 91.5 °C sandwiched between untreated glass; (**f**) schlieren texture of the SmC_A_ phase at 90 °C; (**g**) six-brush dispirations in the schlieren texture of the SmC_A_ phase at 91 °C (200× magnification); (**h**) two-brush defects in the schlieren texture of the SmC_A_ phase at 93.1 °C. The scalebar is 100 μm in all cases.

We first studied **CB8OFFFT** by microscopy confined in a 5 μm cell treated to give planar alignment; on cooling from the isotropic liquid, we observed a wide-temperature nematic phase followed by a short temperature range N_TB_ phase (Figure 1a). Further cooling of the material exhibited a smectic phase, which exhibited a number of striking optical textures, which we will now describe. We observed a parabolic texture (Figure 1b) along with a rope-like texture (Figure 1c,d), reminiscent of those exhibited by the twist-bend nematic phase, but exhibited by a smectic phase. We were unable to locate homeotropic or schlieren regions in the cell, and so we next studied **CB8OFFFT** by microscopy on untreated glass; the nematic phase gave a characteristic *schlieren* texture (Supplementary Materials, Figure S1a) whereas the N_TB_ phase afforded rope-like and blocky textures (Supplementary Materials, Figure S1b).

In the smectic phase, we observed focal conic defects with striations (Figure 1e), a texture classically associated with the helical ferro- and ferrielectric smectic C phases [55]. In the present case, the aperiodicity of the striations suggests their origin is more likely to be tilt domains within the SmC phase rather than the phase being the helical twist-bend type smectic reported recently [56,57,58]. We obtained a schlieren texture for the SmC phase by mechanically shearing the N_TB_ phase just above the transition into the SmC phase to give a homeotropically aligned sample; upon cooling into the SmC phase, we obtained a schlieren texture, with representative photomicrographs given in Figure 1f–h. In the schlieren texture, we observed two-, four- and even six- brush dispirations; such behaviour is diagnostic for the anticlinic phase structures, and thus we conclude this phase to be of the subtype SmC_A_ [59,60,61].

**Figure 2:**
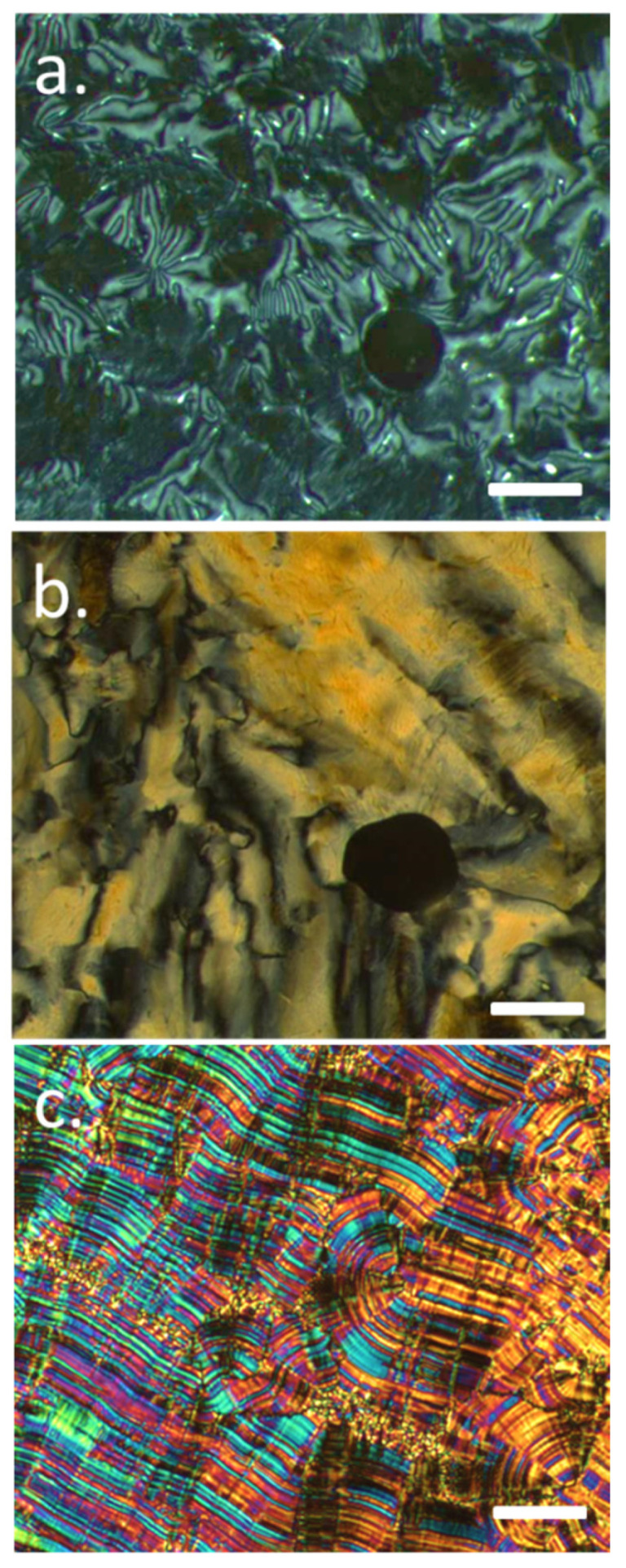
Photomicrographs of **CB8OFFFT**: (**a**) the schlieren texture of the SmC_A_ phase at 85 °C; (**b**) a paramorphotic mosaic texture of the ‘X’ phase at 80 °C, note that this is the same region of the sample as shown in (**a**); (**c**) the banded fan texture of the ‘’X’’ phase at 79 °C. The scale bar corresponds to 50 μm.

Upon supercooling the material, a further phase transition occurred with a large associated enthalpy (ΔH = 7.6 kJ mol^−1^), indicative of the formation of a higher ordered smectic or soft-crystalline phase. When observed by microscopy regions that exhibited a schlieren texture in the SmC_A_ phase yield a broken mosaic texture (Figure 2b). The lack of a schlieren texture in the ‘’X’’ phase ruled out tilted hexatic phases (SmI, SmF); instead, the optical textures were perhaps closest to the soft crystal smectic phases Smectic G phase. Regions exhibiting a fan texture in the SmC_A_ phase gave a banded fan texture, which exhibited long-range out-of-plane correlations and in-plane defects (Figure 2c). These observations contrast with classical LC phases such as B or E, which tended to give continuous domains within the plane.

### 2.2. X-Ray Scattering

**Figure 3:**
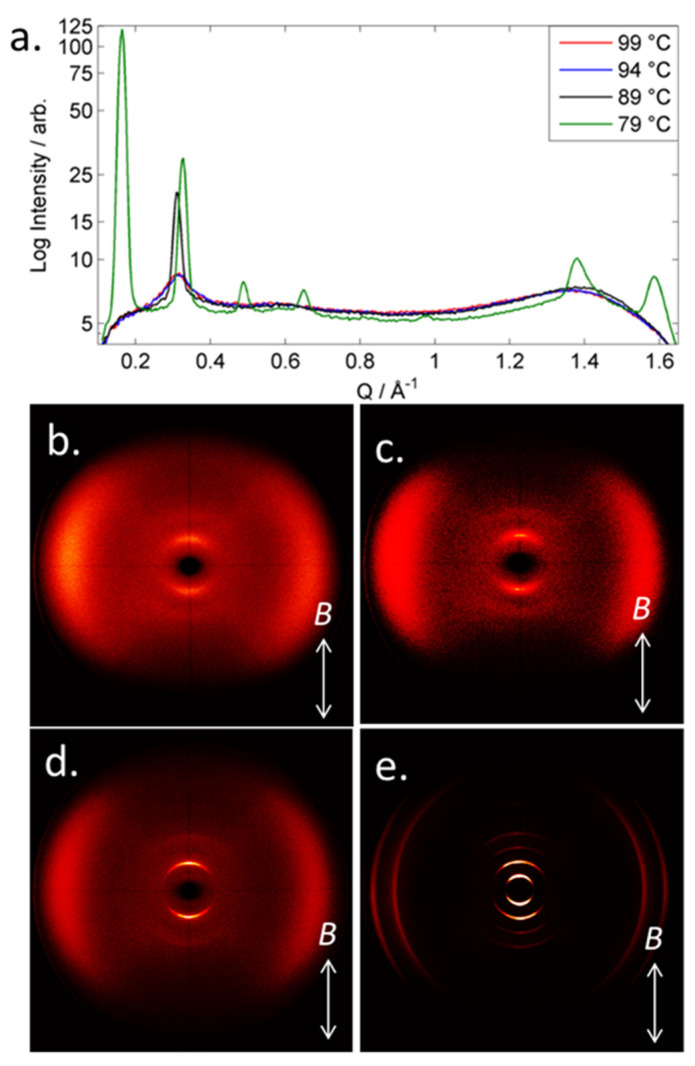
SAXS/WAXS studies on compound **CB8OFFFT**: (**a**) plot of intensity (log, arb.) as a function of Q (Å^−1^) at 99 °C (nematic, red), 94 °C (N_TB_, blue), 89 °C (SmC_A_, black) and 79 °C (X, green). Magnetically aligned 2D SAXS patterns in (**b**) the nematic phase at 105 °C, (**c**) the N_TB_ phase at 94 °C, (**d**) the SmC_A_ phase at 85 °C and (**e**) the ‘’X’’ phase at 74 °C.

We next studied **CB8OFFFT** by SAXS/WAXS; as shown in Figure 3a, both nematic and N_TB_ phases exhibited only diffuse scattering at small angles, whereas the SmC_A_ phase exhibited Bragg scattering due to its layered structure. A highly diffuse second order scattering peak (200) can be seen in the nematic (Figure 3b), N_TB_ (Figure 3c) and SmC_A_ (Figure 3d) phases at Q ~0.6 Å^−1^. All three phases exhibited diffuse scattering at wide angles, indicating a lack of in-plane ordering, whereas the ‘’X’’ phase exhibited a SAXS/WAXS pattern consistent with a highly ordered soft crystal phase (Figure 3e).

Across the SmC_A_-N_TB_ transition, there was no change in the d-spacing of the small angle peak, and the alignment of the X-ray scattering densities were orthogonal to one another, suggesting that there was no change in the overall tilting of the molecules and that the conical angle of the N_TB_ phase became the tilt angle of the SmC_A_ phase. We determined the molecular length—defined here the distance from the nitrogen of the cyanobiphenyl to the 4-fluoro atom of the trifluoroterphenyl unit—to be 32.9 Å at the B3LYP/6-31G(d,p) level of DFT. The layer spacing, which remained constant in the SmC_A_ phase, was found to be 17.4 Å, equal to 0.52 molecular lengths and therefore demonstrating intercalation, with the relative arms of the dimers being mixed in the layer planes. The wide-angle spacing corresponded to the average lateral molecular separation and took practically identical values in the nematic and N_TB_ phases (Q = 1.384 Å^−1^, d = 4.53 Å^−1^) and decreased marginally at the transition to the SmC_A_ phase (Q = 1.399 Å^−1^, d = 4.55 Å^−1^).

**Figure 4:**
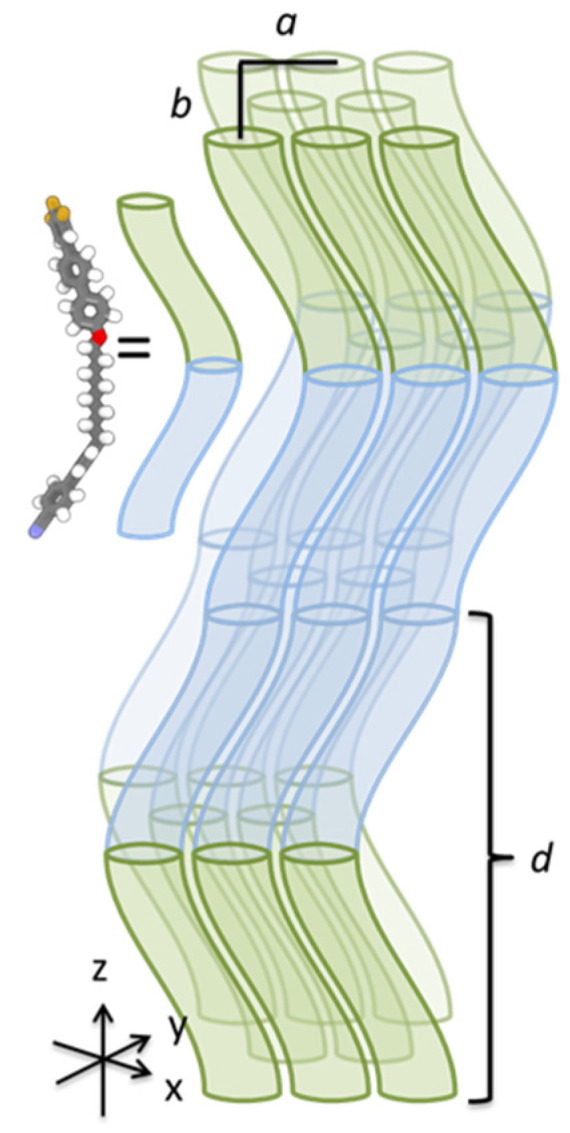
Proposed packing in the ‘’X’’ phase exhibited by **CB8OFFFT**: *d* is the layer spacing (32.9 Å), and a/b correspond to the lateral separations (4.0 Å and 4.6 Å).

In the ‘’X’’ phase, we observed several peaks at small angles (Figure 4e), indicating long-range out-of-plane order, and we indexed these as the 001 through to 005 peaks. The layer spacing, determined from the 001 peak, was approximately equal to the fully extended molecular length. This change in layer spacing in the ‘’X’’ phase was approximately double that of the SmC_A_ phase, indicating that the lower temperature phase lacked the intercalated layer structure seen in the higher temperature phase but had a nanosegregated structure of separated cyanobiphenyl and 3,4,5-trifluoroterphenyl arms. From the scattering vectors (Table 2), we can clearly ascertain that the ‘’X’’ phase was layered rather than cubic or hexagonal. We excluded the possibility of further peaks at smaller angles by increasing the sample-to-detector distance from 121 mm (simultaneous SAXS/WAXS, Q_min_ ≈ 0.1 Å^−1^) to 300 mm (SAXS only, Q_min_ ≈ 0.05 Å^−1^, see Figure S2 in the Supplementary Materials); however, no additional scattering was seen at low Q.

Two Bragg peaks at wide angles (Q = 1.381 Å^−1^ and Q = 1.587 Å^−1^, corresponding to d = 4.6 Å and d = 4.0 Å) indicate orthorhombic local packing within the layers. The lattice parameters are *a* = 33.1 Å, *b* = 8.0 Å and *c*= 4.6 Å. We consider it likely that the ‘’X’’ phase has an anticlinic layer organisation: a synclinic tilt organisation would require the energetically favourable antiparallel cyanobiphenyl-cyanobiphenyl pairing to be overcome, while an orthogonal phase is strongly disfavoured by the bent molecular shape (see Conformational Distributions) and the preceding SmC_A_ phase. The out-of-plane correlation length (determined from the FWHM of the 001 peak) in the X-phase is over 125 nm, with the in-plane correlation lengths being around 20 nm; such values are consistent with a soft-crystalline layered phase with extensive positional order. We observed essentially identical optical textures and SAXS/WAXS patterns for a structurally dissimilar dimer bearing one nitrile and one alkyl terminal group [42], and in two oligomeric materials [62], and we consider it likely these separate observations are of the same phase. The ‘’X’’ phase is therefore analogous to a K or H phase, or possibly a J or G phase, but with additional anticlinic layer organisation. From the proposed structure in Figure 4, it is possible to envisage helical modifications of the ‘’X’’ phase.

Azimuthal integration of the WAXS peak enables calculation of orientational order parameters in the nematic and N_TB_ phases; the partial loss of alignment in the SmC_A_ phase —and total loss of alignment in the X phase—means we were unable to perform this analysis across the full temperature range. Data are presented in the SI accompanying this article (Supplementary Materials, Figure S3); we observed the first three even <P_N_> order parameters to take typical values across the nematic phase range, decreasing at temperatures below the N-N_TB_ transition with the onset of helix formation.

### 2.3. Conformational Distributions

As discussed in the introduction, the N_TB_ phase is largely a consequence of gross molecular shape rather than a specific combination of chemical structural features, and for a given family of materials a linear relationship is often found between T_TB-N_ and T_N-Iso_; indeed, **CB8OFFFT** obeys the relationship we described previously [27].

**Figure 5:**
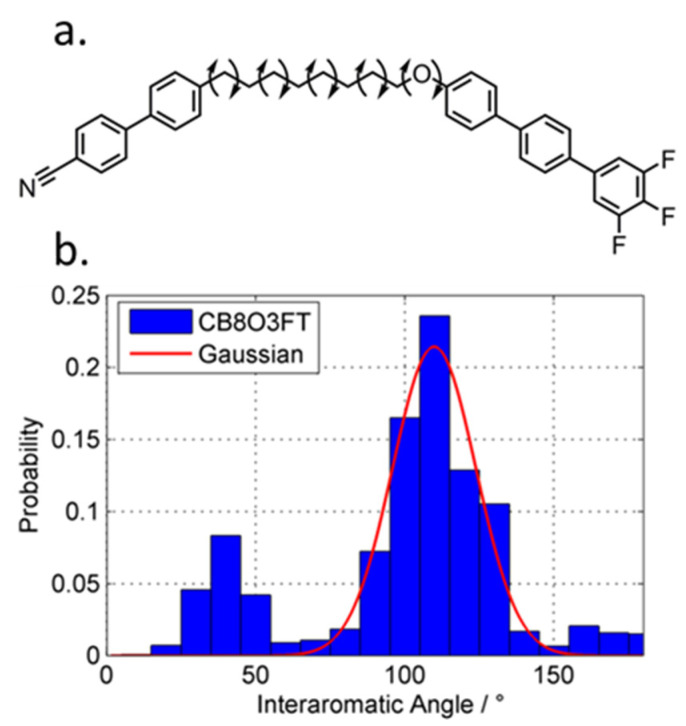
Conformational studies on an isolated molecule: (**a**) the structure of **CB8OFFFT**, where arrows are used to indicate bonds allowed to undergo threefold rotation (twofold for Ph-O); (**b**) histogram plot of the probability of a given interaromatic angle with a Gaussian fit to the major (bent) peaks.

The conformational landscape of an isolated molecule of **CB8OFFFT** was studied via the method described by Archbold [27,31]. We performed fully relaxed scans using the AM1 semi empirical method via the MODREDUNDANT keyword in Gaussian G09 to give a library of conformers. Each dihedral in the spacer was allowed to undergo threefold rotation to give −gauche/trans/+gauche states; the phenyl-oxygen bond was allowed to undergo twofold rotation, while phenyl-phenyl torsions were neglected to reduce the number of conformers (Figure 5a). We extracted Cartesian coordinates and final energies for each conformer; conformers whose energy was above the global minima by 20 kJ mol^−1^ or more were discarded. We calculated the interaromatic angle (i.e., the bend angle) from the mass inertia axes of each rod-like unit. From the energy of each conformation, we obtained a Boltzmann distribution, giving the plot of probability of a given angle, presented in Figure 5. The majority of conformers had bend angles in the range 90–130°, although a minor population of hairpin conformers (<50°) also existed. The FWHM of a Gaussian fit to the major peak was 30°. We calculated the probability weighted average bend angle of **CB8OFFFT** to be 105°; unsurprisingly, both this and the FWHM of the major peak were essentially the same as the analogous material CB8OCB (104°, 34° FWHM). Despite the similarities in conformer distributions, **CB8OFFFT** exhibited two phases (SmC_A_ and X) not observed in the parent material CB8OCB or its analogues [27].

The formation of smectic phases from the N_TB_ phase appeared to be strongly dependent upon the chemical makeup of the mesogenic units, and not simply a consequence of shape, similar to the formation of smectic phases directly from the nematic phase of dimers [63]. None of the presently known structural analogues of **CB8OFFFT** exhibited either SmC_A_ or X phases [27], suggesting that—unlike the bend-driven N_TB_ phase—it is the choice of mesogenic units that dictates the formation of these phases.

## 3. Materials and Methods

### 3.1. Chemical Synthesis

**Scheme 1:**
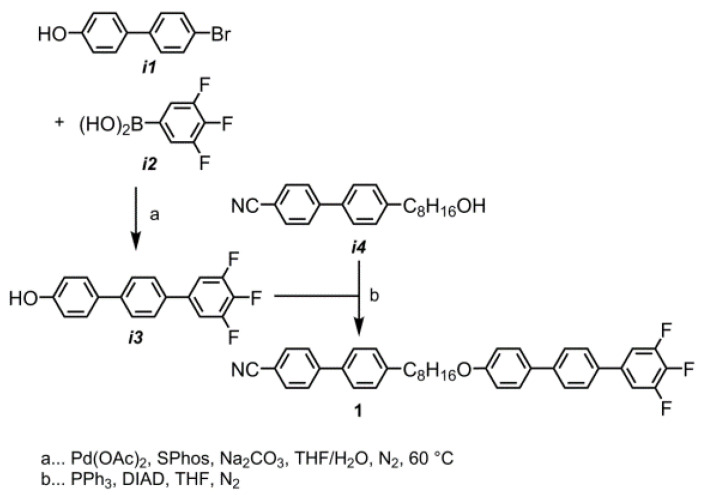


**CB8OFFFT** was prepared by Mitsunobu etherification of CB8OH (***i4***), reported previously [27,64], with 4-hydroxy-3′′,4′′,5′′-trifluoroterphenyl (***i3***), itself prepared from 3,4,5-trifluorobenzene boronic acid (***i2***) and 4-bromo-4′-hydroxybiphenyl (***i1***), as shown in Scheme 1. Full chemical characterisation data for **CB8OOFFT** and intermediate ***i3*** are given in the Supplementary Materials, along with details of the instrumentation used in this work.

### 3.2. Electronic Structure Calculations

Computational chemistry was performed in Gaussian G09 rev D01 [65] on either the YARCC or Viking machines at the University of York. Further details are provided in the SI to this article.

## 4. Conclusions

We report on a novel LC dimer (**CB8OFFFT**) that exhibited the phase sequence N-TB-SmC_A_-‘’X’’, where ‘’X’’ is a soft crystal phase. By combining an appropriately bent central spacer (~105°, FWHM 35°) with mesogenic units with differing polarities and aspect ratios (length/width; CB = 2.3, FFFT = 3.0), we were able to obtain a material that exhibits both N_TB_ and smectic phases. We studied the SmC_A_ phase of **CB8OFFFT** by microscopy, SAXS/WAXS: both supported the assignment as a tilted smectic phase with anticlinc organization and intercalated structure. A logical direction of future study is to attempt to understand why some materials form a helical smectic C phase (SmC_TB_) and others form non-helical smectic C phases. A detailed study of the ‘’X’’ phase by optical microscopy and SAXS/WAXS demonstrated this phase to be soft-crystalline, with an in-plane orthorhombic (or alternatively, monoclinic) packing and long (>100 nm) out of plane correlation lengths. We therefore conclude this phase, which has been observed in a handful of materials to date, is a soft-crystal phase with anticlinic layer organisation.

## Figures and Tables

**Table 1:** Molecular structure (top), transition temperatures (T, °C) and associated enthalpies of transition [ΔH, kJ mol^−1^] for **CB8OFFFT**, determined by differential scanning calorimetry (DSC) at a heat/cool rate of 10 °C min^−1^. Transition temperatures were recorded on the heating cycle with the exception of the monotropic X-SmC_A_ transition, which was recorded on cooling. Uncertainty in our DSC measurements is estimated to be ±0.1 °C and ±0.05 j g^−1^, corresponding to ~0.03 kJ mol^−1^.

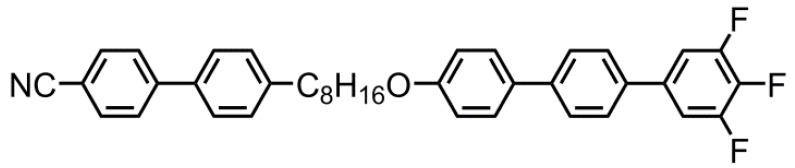					
	MP	X-SmC_A_	SmC_A_-N_TB_	N_TB_-N	N-Iso
T	86.0	81.9	93.4	95.3	145.4
ΔH	8.0	7.6	1.0	0.2	0.6

**Table 2:** Tabulated peak data from SAXS/WAXS of the ‘’X’’ phase of **CB8OFFFT** at a temperature of 74 °C (T/T_N-Iso_ = 0.83).

Peak	Q (Å^−1^)	d
001	0.190 ± 0.002	33.1 ± 0.7
002	0.379 ± 0.003	16.6 ± 0.3
003	0.578 ± 0.005	11.1 ± 0.2
004	0.754 ± 0.02	8.3 ± 0.4
005	0.98 ± 0.03	6.4 ± 0.4
110	1.381 ± 0.02	4.6 ± 0.1
200	1.587 ± 0.02	4.0 ± 0.1

## Data Availability

Data is available from the University of York data archive.

## Supplementary Materials

The following are available online. Figure S1: “ POM images (crossed polarisers, ×100 magnification, scale bar = 50 μm) of (a) the nematic phase of CB8OFFFT at 107 °C, (b) the NTB phase of CB8OFFFT at 92 °C, (c) the SmCA phase of CB8OFFFT at 91.2 °C, (d) the ‘X’ phase of CB8OFFFT at 60 °C”, Figure S2: “SAXS studies on CB8OFFFT: (a) plot of log. intensity (arb.) as a function of Q (Å-1) at 99 °C (nematic, red), 94 °C (NTB, blue), 89 °C (SmCA, black), 78 °C (X, green); (b) magnetically aligned 2D SAXS patterns in the nematic phase at 105 °C (b), the NTB phase at 94 °C (c) the SmCA phase at 89 °C (d), the X phase at 78 °C (e)”; Figure S3: “Plot of the orientational order parameters of CB8OFFFT as a function of reduced temperature.”

## Sample Availability

Samples of the compounds are available from R.J.M. or J.W.G. upon reasonable request.

## References

[1] Dozov I. (2001). On the spontaneous symmetry breaking in the mesophases of achiral banana-shaped molecules. Europhys. Lett..

[2] Cestari M., Diez-Berart S., Dunmur D.A., Ferrarini A., de la Fuente M.R., Jackson D.J., Lopez D.O., Luckhurst G.R., Perez-Jubindo M.A., Richardson R.M. (2011). Phase behavior and properties of the liquid-crystal dimer 1″,7″-bis(4-cyanobiphenyl-4′-yl) heptane: A twist-bend nematic liquid crystal. Phys. Rev. E Stat. Nonlin Soft Matter Phys..

[3] Mertelj A., Cmok L., Sebastian N., Mandle R.J., Parker R.R., Whitwood A.C., Goodby J.W., Copic M. (2018). Splay nematic phase. Phys. Rev. X.

[4] Zhu C., Tuchband M.R., Young A., Shuai M., Scarbrough A., Walba D.M., Maclennan J.E., Wang C., Hexemer A., Clark N.A. (2016). Resonant carbon k-edge soft x-ray scattering from lattice-free heliconical molecular ordering: Soft dilative elasticity of the twist-bend liquid crystal phase. Phys. Rev. Lett..

[5] Chen D., Porada J.H., Hooper J.B., Klittnick A., Shen Y., Tuchband M.R., Korblova E., Bedrov D., Walba D.M., Glaser M.A. (2013). Chiral heliconical ground state of nanoscale pitch in a nematic liquid crystal of achiral molecular dimers. Proc. Natl. Acad. Sci. USA.

[6] Borshch V., Kim Y.K., Xiang J., Gao M., Jakli A., Panov V.P., Vij J.K., Imrie C.T., Tamba M.G., Mehl G.H. (2013). Nematic twist-bend phase with nanoscale modulation of molecular orientation. Nat. Commun..

[7] Gorecka E., Vaupotic N., Zep A., Pociecha D., Yoshioka J., Yamamoto J., Takezoe H. (2015). A twist-bend nematic (n-tb) phase of chiral materials. Angew. Chem. Int. Ed..

[8] Mandle R.J., Goodby J. (2018). Optically active bimesogens incorporating branched central spacers. Rsc. Adv..

[9] Walker R., Pociecha D., Storey J.M.D., Gorecka E., Imrie C.T. (2019). The chiral twist-bend nematic phase (n*tb). Chem. A Eur. J..

[10] Mandle R.J., Davis E.J., Archbold C.T., Cowling S.J., Goodby J.W. (2014). Microscopy studies of the nematic ntbphase of 1,11-di-(1′′-cyanobiphenyl-4-yl)undecane. J. Mater. Chem. C.

[11] Stevenson W.D., Ahmed Z., Zeng X.B., Welch C., Ungar G., Mehl G.H. (2017). Molecular organization in the twist-bend nematic phase by resonant x-ray scattering at the se k-edge and by saxs, waxs and gixrd. Phys. Chem. Chem. Phys..

[12] Robles-Hernandez B., Sebastian N., de la Fuente M.R., Lopez D.O., Diez-Berart S., Salud J., Ros M.B., Dunmur D.A., Luckhurst G.R., Timimi B.A. (2015). Twist, tilt, and orientational order at the nematic to twist-bend nematic phase transition of 1″,9 ″-bis(4-cyanobiphenyl-4′-yl) nonane: A dielectric, h-2 nmr, and calorimetric study. Phys. Rev. E.

[13] Emsley J.W., Lesot P., Luckhurst G.R., Meddour A., Merlet D. (2013). Chiral solutes can seed the formation of enantiomorphic domains in a twist-bend nematic liquid crystal. Phys. Rev. E.

[14] Emsley J.W., Lelli M., Lesage A., Luckhurst G.R. (2013). A comparison of the conformational distributions of the achiral symmetric liquid crystal dimer cb7cb in the achiral nematic and chiral twist-bend nematic phases. J. Phys. Chem. B.

[15] Jokisaari J.P., Luckhurst G.R., Timimi B.A., Zhu J.F., Zimmermann H. (2015). Twist-bend nematic phase of the liquid crystal dimer cb7cb: Orientational order and conical angle determined by xe-129 and h-2 nmr spectroscopy. Liq. Cryst..

[16] Zhang Z.P., Panov V.P., Nagaraj M., Mandle R.J., Goodby J.W., Luckhurst G.R., Jones J.C., Gleeson H.F. (2015). Raman scattering studies of order parameters in liquid crystalline dimers exhibiting the nematic and twist-bend nematic phases. J. Mater. Chem. C.

[17] Meyer C., Blanc C., Luckhurst G.R., Davidson P., Dozov I. (2020). Biaxiality-driven twist-bend to splay-bend nematic phase transition induced by an electric field. Sci. Adv..

[18] Challa P.K., Borshch V., Parri O., Imrie C.T., Sprunt S.N., Gleeson J.T., Lavrentovich O.D., Jakli A. (2014). Twist-bend nematic liquid crystals in high magnetic fields. Phys. Rev. E.

[19] Mandle R.J. (2016). The dependency of twist-bend nematic liquid crystals on molecular structure: A progression from dimers to trimers, oligomers and polymers. Soft Matter.

[20] Mandle R.J., Goodby J.W. (2016). A liquid crystalline oligomer exhibiting nematic and twist-bend nematic mesophases. Chemphyschem.

[21] Arakawa Y., Komatsu K., Inui S., Tsuji H. (2020). Thioether-linked liquid crystal dimers and trimers: The twist-bend nematic phase. J. Mol. Struct..

[22] Walker R., Pociecha D., Crawford C.A., Storey J.M.D., Gorecka E., Imrie C.T. (2020). Hydrogen bonding and the design of twist-bend nematogens. J. Mol. Liq..

[23] Walker R. (2020). The twist-bend phases: Structure–property relationships, chirality and hydrogen-bonding. Liq. Cryst. Today.

[24] Chen D., Nakata M., Shao R., Tuchband M.R., Shuai M., Baumeister U., Weissflog W., Walba D.M., Glaser M.A., Maclennan J.E. (2014). Twist-bend heliconical chiral nematic liquid crystal phase of an achiral rigid bent-core mesogen. Phys. Rev. E Stat. Nonlin Soft Matter Phys..

[25] Mandle R.J., Goodby J.W. (2018). A nanohelicoidal nematic liquid crystal formed by a non-linear duplexed hexamer. Angew. Chem. Int. Ed..

[26] Mandle R.J., Goodby J.W. (2016). Does topology dictate the incidence of the twist-bend phase? Insights gained from novel unsymmetrical bimesogens. Chem. Eur. J..

[27] Pocock E.E., Mandle R.J., Goodby J.W. (2018). Molecular shape as a means to control the incidence of the nanostructured twist bend phase. Soft Matter.

[28] Lesac A., Baumeister U., Dokli I., Hameršak Z., Ivšić T., Kontrec D., Viskić M., Knežević A., Mandle R.J. (2018). Geometric aspects influencing n-ntb transition - implication of intramolecular torsion. Liq. Cryst..

[29] Knežević A., Sapunar M., Buljan A., Dokli I., Hameršak Z., Kontrec D., Lesac A. (2018). Fine-tuning the effect of π–π interactions on the stability of the ntb phase. Soft Matter.

[30] Mandle R.J., Archbold C.T., Sarju J.P., Andrews J.L., Goodby J.W. (2016). The dependency of nematic and twist-bend mesophase formation on bend angle. Sci. Rep. Uk.

[31] Archbold C.T., Mandle R.J., Andrews J.L., Cowling S.J., Goodby J.W. (2017). Conformational landscapes of bimesogenic compounds and their implications for the formation of modulated nematic phases. Liq. Cryst..

[32] Greco C., Luckhurst G.R., Ferrarini A. (2014). Molecular geometry, twist-bend nematic phase and unconventional elasticity: A generalised maier-saupe theory. Soft Matter.

[33] Vaupotic N., Cepic M., Osipov M.A., Gorecka E. (2014). Flexoelectricity in chiral nematic liquid crystals as a driving mechanism for the twist-bend and splay-bend modulated phases. Phys. Rev. E.

[34] Arakawa Y., Ishida Y., Tsuji H. (2020). Ether- and thioether-linked naphthalene-based liquid-crystal dimers: Influence of chalcogen linkage and mesogenic-arm symmetry on the incidence and stability of the twist-bend nematic phase. Chem. A Eur. J..

[35] Arakawa Y., Komatsu K., Tsuji H. (2019). Twist-bend nematic liquid crystals based on thioether linkage. New J. Chem..

[36] Mandle R.J., Davis E.J., Archbold C.T., Voll C.C.A., Andrews J.L., Cowling S.J., Goodby J.W. (2015). Apolar bimesogens and the incidence of the twist-bend nematic phase. Chem. Eur. J..

[37] Forsyth E., Paterson D.A., Cruickshank E., Strachan G.J., Gorecka E., Walker R., Storey J.M.D., Imrie C.T. (2020). Liquid crystal dimers and the twist-bend nematic phase: On the role of spacers and terminal alkyl chains. J. Mol. Liq..

[38] Panov V.P., Varney M.C.M., Smalyukh I.I., Vij J.K., Tamba M.G., Mehl G.H. (2015). Hierarchy of periodic patterns in the twist-bend nematic phase of mesogenic dimers. Mol. Cryst. Liq. Cryst..

[39] Ivšić T., Baumeister U., Dokli I., Mikleušević A., Lesac A. (2017). Sensitivity of the ntb phase formation to the molecular structure of imino-linked dimers. Liq. Cryst..

[40] Mandle R.J., Davis E.J., Lobato S.A., Vol C.C., Cowling S.J., Goodby J.W. (2014). Synthesis and characterisation of an unsymmetrical, ether-linked, fluorinated bimesogen exhibiting a new polymorphism containing the n(tb) or ‘twist-bend’ phase. Phys. Chem. Chem. Phys..

[41] Mandle R.J., Goodby J.W. (2016). A twist-bend nematic to an intercalated, anticlinic, biaxial phase transition in liquid crystal bimesogens. Soft Matter.

[42] Mandle R.J., Goodby J.W. (2016). Intercalated soft-crystalline mesophase exhibited by an unsymmetrical twist-bend nematogen. Crystengcomm.

[43] Mandle R.J., Cowling S.J., Goodby J.W. (2017). Combined microscopy, calorimetry and x-ray scattering study of fluorinated dimesogens. Sci. Rep.-Uk.

[44] Knezevic A., Dokli I., Sapunar M., Segota S., Baumeister U., Lesac A. (2018). Induced smectic phase in binary mixtures of twist-bend nematogens. Beilstein J. Nanotech..

[45] Sepelj M., Lesac A., Baumeister U., Diele S., Nguyen H.L., Bruce D.W. (2007). Intercalated liquid-crystalline phases formed by symmetric dimers with an alpha, omega-diiminoalkylene spacer. J. Mater. Chem..

[46] Al-Janabi A., Mandle R.J. (2020). Utilising saturated hydrocarbon isosteres of para benzene in the design of twist-bend nematic liquid crystals. Chemphyschem.

[47] Ahmed Z., Welch C., Mehl G.H. (2015). The design and investigation of the self-assembly of dimers with two nematic phases. Rsc. Adv..

[48] Mandle R.J., Goodby J.W. (2016). Progression from nano to macro science in soft matter systems: Dimers to trimers and oligomers in twist-bend liquid crystals. Rsc. Adv..

[49] Ivsic T., Vinkovic M., Baumeister U., Mikleusevic A., Lesac A. (2016). Towards understanding the n-tb phase: A combined experimental, computational and spectroscopic study. Rsc. Adv..

[50] Abberley J.P., Jansze S.M., Walker R., Paterson D.A., Henderson P.A., Marcelis A.T.M., Storey J.M.D., Imrie C.T. (2017). Structure–property relationships in twist-bend nematogens: The influence of terminal groups. Liq. Cryst..

[51] Leadbetter A.J., Frost J.C., Gaughan J.P., Gray G.W., Mosley A. (1979). The structure of smectic a phases of compounds with cyano end groups. J. Phys. Fr..

[52] Kirsch P., Bremer M. (2000). Nematic liquid crystals for active matrix displays: Molecular design and synthesis. Angew. Chem. Int. Ed..

[53] Goodby J.W. (2011). The nanoscale engineering of nematic liquid crystals for displays. Liq. Cryst..

[54] Paterson D.A., Abberley J.P., Harrison W.T., Storey J.M., Imrie C.T. (2017). Cyanobiphenyl-based liquid crystal dimers and the twist-bend nematic phase. Liq. Cryst..

[55] Kasthuraiah N., Sadashiva B.K., Krishnaprasad S., Nair G.G. (1996). Ferroelectric and antiferroelectric liquid crystalline phases in some pyridine carboxylic acid derivatives. J. Mater. Chem..

[56] Tamba M.G., Salili S.M., Zhang C., Jakli A., Mehl G.H., Stannarius R., Eremin A. (2015). A fibre forming smectic twist-bent liquid crystalline phase. Rsc. Adv..

[57] Sreenilayam S.P., Panarin Y.P., Vij J.K., Panov V.P., Lehmann A., Poppe M., Prehm M., Tschierske C. (2016). Spontaneous helix formation in non-chiral bent-core liquid crystals with fast linear electro-optic effect. Nat. Commun..

[58] Abberley J.P., Killah R., Walker R., Storey J.M.D., Imrie C.T., Salamonczyk M., Zhu C.H., Gorecka E., Pociecha D. (2018). Heliconical smectic phases formed by achiral molecules. Nat. Commun..

[59] Takanishi Y., Takezoe H., Fukuda A., Komura H., Watanabe J. (1992). Simple method for confirming the antiferroelectric structure of smectic liquid crystals. J. Mater. Chem.

[60] Elston S.J., Sambles J.R. (1998). The Optics of Thermotropic Liquid Crystals.

[61] Cowling S.J., Davis E.J., Mandle R.J., Goodby J.W. (2013). Defect textures of liquid crystals. Progress in Liquid Crystal Science and Technology.

[62] Mandle R.J., Stevens M.P., Goodby J.W. (2017). Developments in liquid-crystalline dimers and oligomers. Liq. Cryst..

[63] Goodby J.W. (2017). Free volume, molecular grains, self-organisation, and anisotropic entropy: Machining materials. Liq. Cryst..

[64] Wang K., Jirka M., Rai P., Twieg R.J., Szilvási T., Yu H., Abbott N.L., Mavrikakis M. (2018). Synthesis and properties of hydroxy tail-terminated cyanobiphenyl liquid crystals. Liq. Cryst..

[65] (2009). Gaussian, Version 09, Revision D.01.

