# Supplementary material for: Experimental and Computational Study of a Liquid Crystalline Dimesogen Exhibiting Nematic, Twist-Bend Nematic, Intercalated Smectic, and Soft Crystalline Mesophases
Source: Molecules. 2021 Jan 20;26(3):532. doi: 10.3390/molecules26030532 (PMC7864162; doi:10.3390/molecules26030532)
Supplement: Supplementary file 1 [file molecules-26-00532-s001.pdf]

1. Methods

1.1. General Techniques

Miscellaneous solvents were purchased from Fisher Scientific dried by sequential percolation through columns of activated alumina and copper Q5 catalyst prior to use. Unless otherwise noted, chemical reagents were purchased from commercial suppliers and used without further purification. The intermediate ***i4*** was prepared as described previously. [1] Reactions were monitored by thin layer chromatography (TLC) using an appropriate solvent system. Silica coated aluminium TLC plates used were purchased from Merck (Kieselgel 60 F-254) and visualised using UV light at wavelengths of both 254 nm and 365 nm. Column chromatography was performed using flash grade silica from Fluorochem (40 - 63μm particle size). Yields refer to chromatographically (HPLC) and spectroscopically (^{1}H NMR, ^{13}C [2] NMR and ^{19}F NMR) homogenous material.

1.2. Nuclear Magnetic Resonance

NMR spectra were recorded on a JEOL ECS spectrometer operating at 400 MHz (^{1}H), 100.5 MHz (^{13}C[2]) and 376.4 MHz (^{19}F NMR) as solutions in deuterated chloroform. Spectra were referenced to the residual protic solvent for ^{1}H (7.26 ppm), ^{13}C[2] to the resonance of $CDCl_3$ (77.16 ppm) and ^{19}F were unreferenced.

1.3. Mass Spectrometry

Mass spectra were recorded on a Bruker compact time of flight mass spectrometer with both ESI and APCI sources, and we extend our gratitude to Mr. Karl Heaton of the University of York for obtaining MS data.

1.4. High Performance Liquid Chromatography

High-performance liquid chromatography was performed on a Shimadzu Prominence modular HPLC system comprising a LC-20A quaternary solvent pump, a DGU-20A$_5$ degasser, a SIL-20A autosampler, a CBM-20A communication bus, a CTO-20A column oven, and a SPO-20A dual wavelength UV-vis detector operating at 220/250 nm. The column used was an Alltech C18 bonded reverse-phase silica column with a 5 μm pore size, an internal diameter of 10 mm and a length of 250 mm. In all cases the mobile phase used was neat acetonitrile, purchased from Fisher Scientific UK.

1.5. Polarised Optical Microscopy

Polarised optical microscopy was performed on a Zeiss Axioskop 40Pol microscope using a Mettler FP82HT hotstage controlled by a Mettler FP90 central processor. Photomicrographs were captured *via* an InfinityX-21 MP digital camera mounted atop the microscope.

1.6. Differential Scanning Calorimetry.

Differential scanning calorimetry was performed on a Mettler DSC822^e fitted with an autosampler operating with Mettler Star^e software and calibrated before use against an indium standard (onset = 156.55 ± 0.2 °C, ΔH = 28.45 ± 0.40 Jg^{-1}) under an atmosphere of dry nitrogen.

1.7. Small Angle X-ray Scattering

Small angle X-ray scattering was performed using a Bruker D8 Discover using copper Kα radiation (λ = 0.154056 nm) from a 1 μS microfocus source. Samples to be studied by SAXS were filled into 0.9 mm I.D. borosilicate glass capillaries (Cappilary Tube Supplies UK) and placed into a custom-built graphite rod furnace with a temperature accuracy of at least +/- 0.1 °C.

Diffraction patterns were recorded on a 2048x2048 pixel Bruker VANTEC 500 area detector set at a distance of 121 mm from the sample. Alignment was afforded with a pair of 1T magnets perpendicular to the incident beam, with the field strength at the sample position being approximately 0.6T.Two-dimensional scattering patterns were collected on cooling from the isotropic liquid until crystallisation in ~ 1.2 °C intervals. Additionally, the 2D SAXS pattern obtained for an unfilled borosilicate glass capillary tube recorded, and later used as the background.

Raw 2D SAXS patterns were processed by subtracting the 2D pattern of an air filled glass capillary; the background subtracted data was separately radially and azimuthally averaged to give scattering intensity as a function of Q and χ respectively. The low angle portion of radially averaged data was fitted with a Voigt function to yield the peak position(s) (i.e. layer spacing). Azimuthally averaged data was used for calculation of the orientational order parameters according to [3] [4]. Data was scaled and exported as .tiff images for presentation in the manuscript.

All data processing was performed using in house developed Matlab scripts and functions which are available from the corresponding author upon reasonable request.

1.8. Electronic Structure Calculations

Computational chemistry was performed in Gaussian G09 rev D01 [5] on either the ARC3 machine at the University of Leeds, the YARCC or Viking machines at the University of York. Post optimisation, a frequency calculation was used to confirm the absence of imaginary frequencies and so confirm the optimised geometries were true minima.

2. Synthetic Scheme

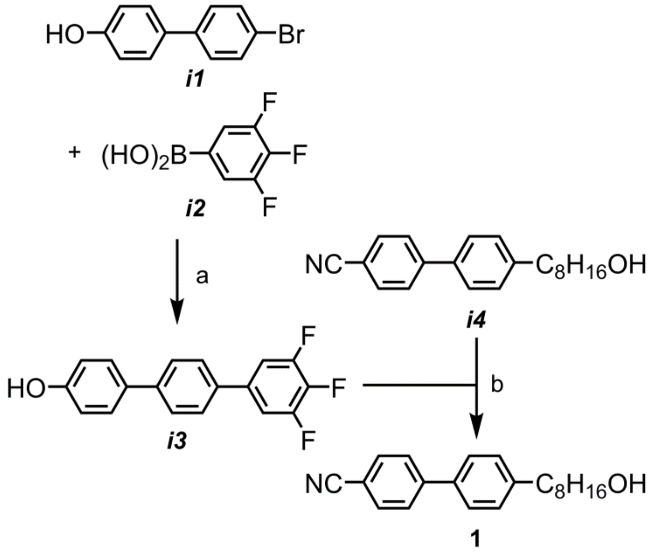

a... Pd(OAc)$_2$, SPhos, Na$_2$CO$_3$, THF/H$_2$O, N$_2$, 60 °C
b... PPh$_3$, DIAD, THF, N$_2$

Scheme 1

3. Chemical Characterisation

3.1. Characterisation of Intermediates

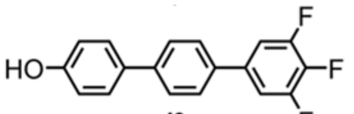

i3

3'',4'',5''-trifluoro-[1,1':4',1''-terphenyl]-4-ol (*i3*)

A solution of 4-hydroxy-4′-bromobiphenyl (**i1**, 13.9 g, 55.7 mmol) in a biphasic mixture of THF (150 ml) and aqueous sodium carbonate (2M, 150 ml) was degassed by sparging with nitrogen under ultrasonic agitation for 30 minutes. The biphasic reaction mixture was heated to reflux and 3,4,5-trifluorobenzene boronic acid (***i2***, 10 g, 56.8 mmol) added as a solid in one portion. The biphasic reaction mixture was heated for 15 minutes before the addition of a solution of degassed solution of $Pd(OAc)_4$ (50 mg) and SPhos (50 mg) in THF (2 ml) in one portion. The reaction was monitored by TLC and was complete within 8 hours, at which point the reaction mixture was allowed to cool to ambient temperature and the biphasic mixture separated. The aqueous layer was washed with MTBE (3 x 40 ml) and discarded. The combined organic extracts were dried over magnesium sulphate and concentrated *in vacuo* to an off white solid. Column chromatography of the crude residue over SiO_2 with 3:1 DCM/hexanes as the eluent (Rf_{DCM} = 0.35), followed by recrystalisation from ethanol, gave the title compound as white plates.

Yield 14.7 g (88%)

^{1}H NMR: 6.84 (2H, ddd, *J* = 1.8 Hz, *J* = 2.8 Hz, *J* = 8.7 Hz, Ar*H*), 7.48 (2H, ddd, *J* = 1.8 Hz, *J* = 2.8 Hz, *J* = 8.7 Hz, Ar*H*), 7.55 – 7.64 (4H, m, Ar*H*), 7.65 – 7.69 (2H, m, Ar*H*), 9.56 (1H, s, ArO*H*)

$^{13}C^{2}$ NMR: 110.70 (dd, *J* = 4.8 Hz, *J* = 16.3 Hz), 115.78, 126.36, 127.09, 127.67, 129.93, 134.71, 136.52 (dt, *J* = 3.8 Hz, *J* = 8.6 Hz), 138.17 (tt, *J* = 15.3 Hz, *J* = 250.2 Hz), 140.32, 150.07 (ddd, *J* = 4.8 Hz, *J* = 12.0 Hz, *J* = 247.3 Hz), 157.48

^{19}F NMR: -163.72 (1F, tt, *J* = 5.8 Hz, *J* = 21.7 Hz, Ar*F*), -134.78 (2F, dd, *J* = 10.1 Hz, *J* = 21.7 Hz, Ar*F*)

MS (ESI+): 301.0851 (calcd. for $C_{18}H_{12}F_3O$: 301.08348, M + H)

3.2. Characterisation of CB8OFFFT

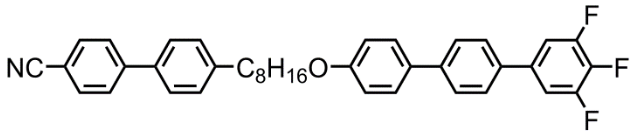

CB8OFFFT: 4'-(8-((3'',4'',5''-trifluoro-[1,1':4',1''-terphenyl]-4-yl)oxy)octyl)-[1,1'-biphenyl]-4-carbonitrile

i4 (100 mg, 0.273 mmol), PPh_3 (1 mmol, 262 mg), and ***i3*** (89.3 mg, 0.287 mmol) were disolved into anhydrous THF (10 ml) under an atmosphere of dry nitrogen. DIAD (1 mmol, 199 µl) was added in one portion and the resulting solution stirred until complete consumption of ***i3*** and ***i4*** (~4 h). The solvent was removed *in vacuo* and the crude reaction mixture purified by flash chromatography with 2:1 DCM/hexanes as the eluent. Recrystalisation of the chromatographed material from ethanol afforded the title compound as fine colourless crystals

Yield: 120 mg (75%)

Rf: 0.72 (DCM)

^{1}H NMR: 1.34 – 1.50 (8H, m, -C<u>H</u>$_2$-), 1.62 – 1.70 (2 H, m, -C<u>H</u>$_2$-), 1.76 – 1.84 (2 H, m, -C<u>H</u>$_2$-), 2.67 (2H, t, *J* = 6.8 Hz, Ar-C<u>H</u>$_2$-CH$_2$), 4.00 (2H, t, *J* = 6.8 Hz, ArO-C<u>H</u>$_2$-CH$_2$-), 6.97 (2H, ddd, *J* = 1.8 Hz, *J* = 2.4 Hz, *J* = 8.8 Hz, Ar<u>H</u>), 7.18 – 7.32 (4H, m, Ar<u>H</u>), 7.47 – 7.58 (6H, m, Ar<u>H</u>), 7.60 – 7.75 (6H, m, Ar<u>H</u>),

^{13}C ^{2} NMR: 26.16, 29.34, 29.38, 29.42, 29.53, 31.50, 35.74, 68.19, 110.92 (dd, *J* = 5.8 Hz, *J* = 15.9 Hz), 115.01, 119.19, 126.73, 127.28, 127.35, 127.43, 127.46 (d, *J* = 5.7 Hz), 127.60, 128.16, 129.32, 132.52, 132.69, 136.42 (d, *J* = 3.0 Hz), 136.59, 137.05, 140.55, 141.03, 143.85, 145.72, 150.55 (ddd, *J* = 4.3 Hz, *J* = 10.2 Hz, *J* = 246.6 Hz),

^{19}F NMR: -162.64 (1F, tt, *J* = 6.3 Hz, *J* = 20.5 Hz, Ar<u>F</u>), -134.07 (2F, dd, *J* = 8.6 Hz, *J* = 20.5 Hz, Ar<u>F</u>)

MS (APCI): 626.415254 (calcd. for $C_{42}H_{54}F_2NO$: 626.416798, M + H)

HPLC: >99.5%

4. Supplementary Photomicrographs

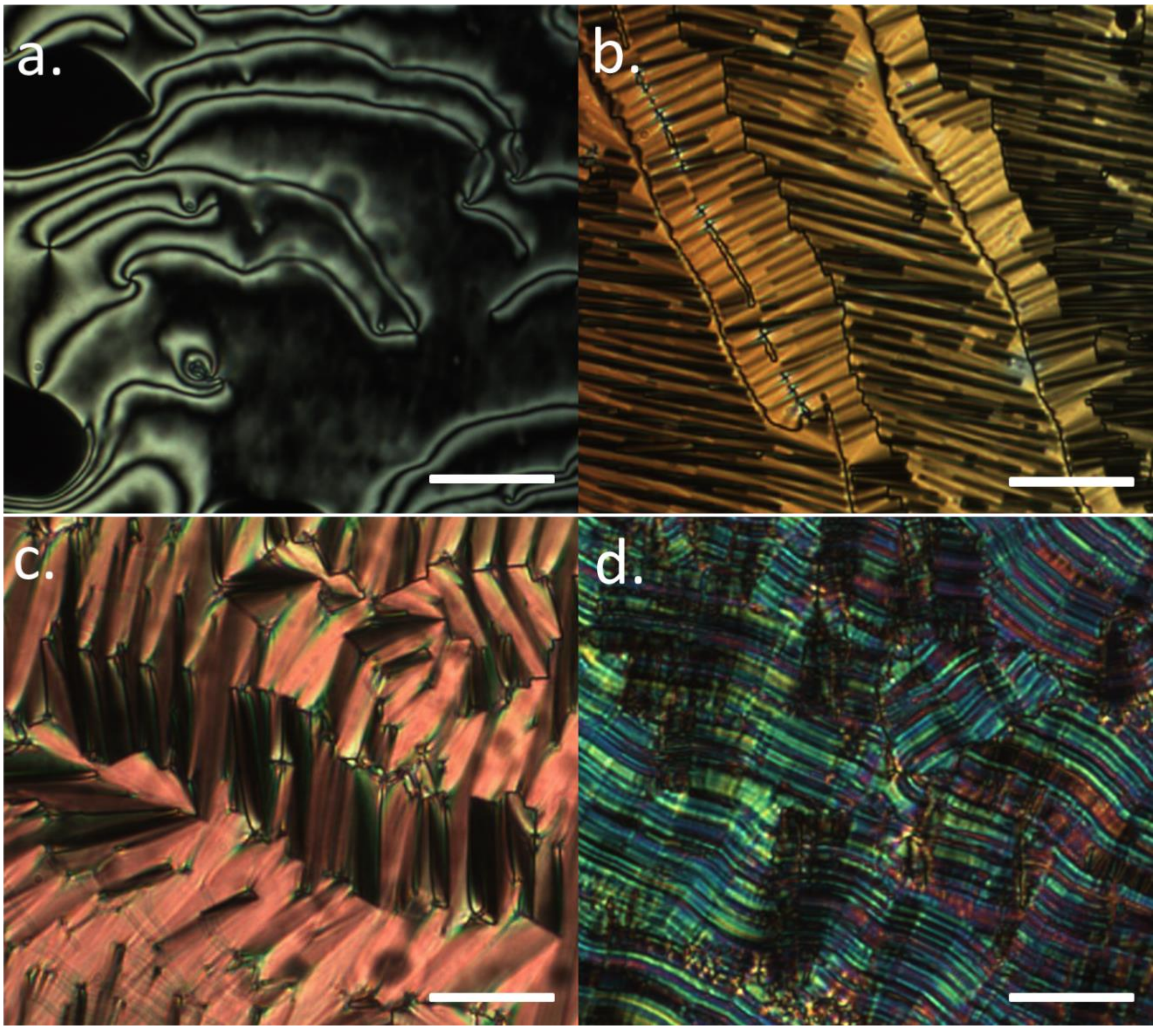

Figure SI-1: POM images (crossed polarisers, x100 magnification, scale bar = 50 μm) of (a) the nematic phase of **CB8OFFFT** at 107 °C, (b) the N_{TB} phase of **CB8OFFFT** at 92 °C, (c) the SmC_A phase of **CB8OFFFT** at 91.2 °C, (d) the 'X' phase of **CB8OFFFT** at 60 °C

5. Supplementary X-ray Scattering Experiments

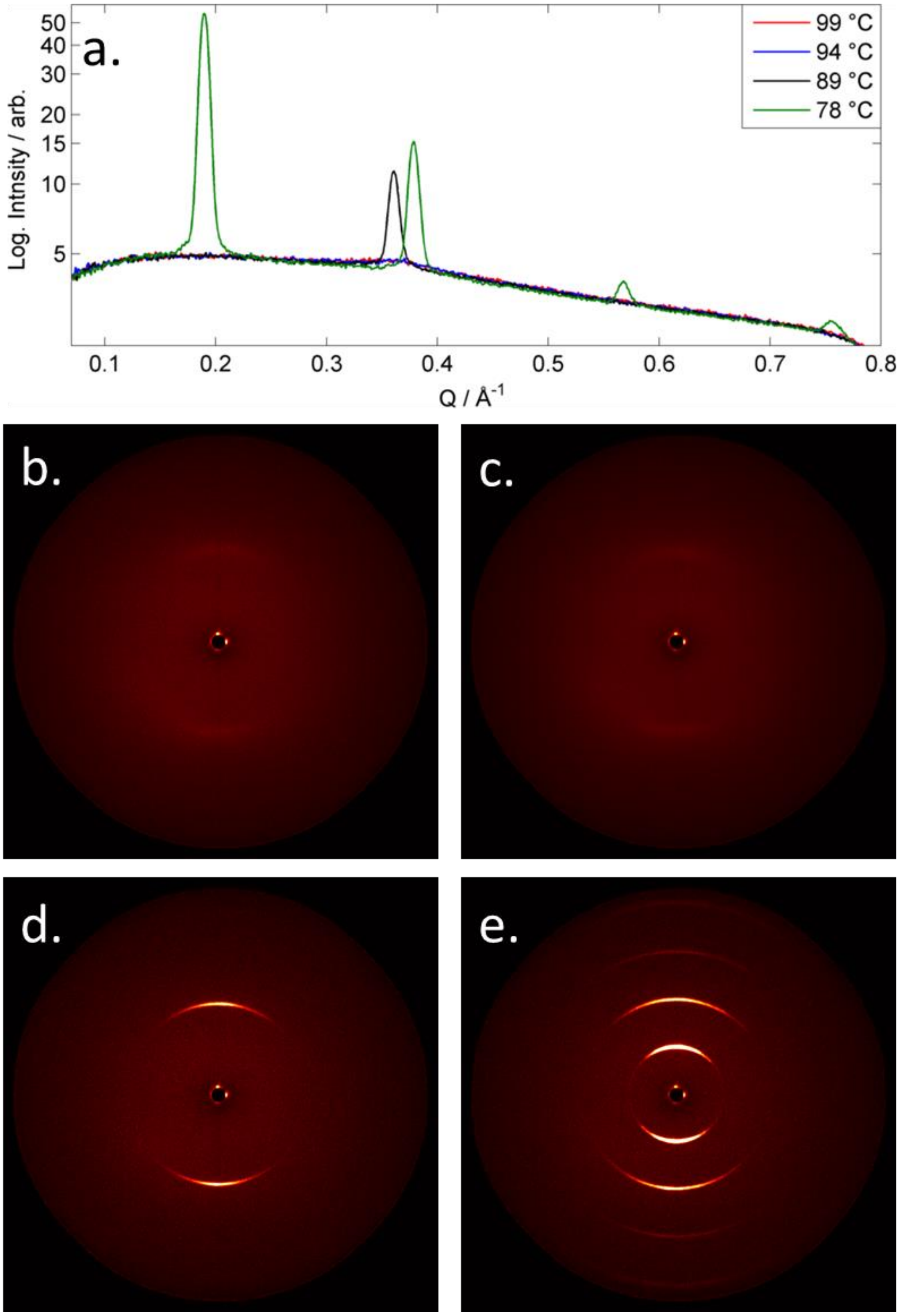

Figure SI-2: SAXS studies on **CB8OFFFT**: (a) plot of log. intensity (arb.) as a function of Q (Å^{-1}) at 99 °C (nematic, red), 94 °C (N_{TB}, blue), 89 °C (SmC$_A$, black), 78 °C (X, green); (b) magnetically aligned 2D SAXS patterns in the nematic phase at 105 °C (b), the N_{TB} phase at 94 °C (c) the SmC$_A$ phase at 89 °C (d), the X phase at 78 °C (e).

The alignment of **CB8OFFFT** during SAXS/WAXS studies was sufficient in the nematic and N_{TB} phases to permit measurement of the orientational order parameters. We used the Kratky method as outlined elsewhere. [3, 4] The alignment of the sample deteriorates following the SmC_A-N_{TB} transition and so order parameters were not calculated below a reduced temperature of 0.876 (93.5 °C). Both $\langle P_2 \rangle$ and $\langle P_4 \rangle$ increase as **CB8OFFFT** is cooled through its nematic range, whereas $\langle P_6 \rangle$ is effectively zero. On entering the N_{TB} phase ($T/T_{N\text{-}Iso}$ ~ 0.88, T = 95.3 °C) all three order parameters decrease, mirroring the behaviour of other systems studied by this method. [6]

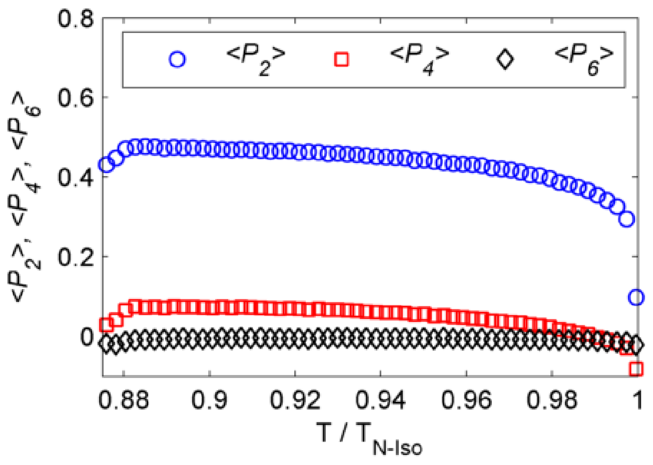

Fig SI-3: Plot of the orientational order parameters of **CB8OFFFT** as a function of reduced temperature.

6. References

1. E. E. Pocock, R. J. Mandle and J. W. Goodby, *Soft Matter*, 2018, **14**, 2508-2514.
2. N. T. Abdel-Ghani, M. F. A. E-Ghar and A. M. Mansour, *Spectrochim Acta A*, 2013, **104**, 134-142.
3. M. T. Sims, L. C. Abbott, R. M. Richardson, J. W. Goodby and J. N. Moore, *Liq Cryst*, 2018, **46**, 11-24.
4. D. M. Agra-Kooijman, M. R. Fisch and S. Kumar, *Liq Cryst*, 2018, **45**, 680-686.
5. M. J. Frisch, G. W. Trucks, H. B. Schlegel, G. E. Scuseria, M. A. Robb, J. R. Cheeseman, G. Scalmani, V. Barone, B. Mennucci, G. A. Petersson, H. Nakatsuji, M. Caricato, X. Li, H. P. Hratchian, A. F. Izmaylov, J. Bloino, G. Zheng, J. L. Sonnenberg, M. Hada, M. Ehara, K. Toyota, R. Fukuda, J. Hasegawa, M. Ishida, T. Nakajima, Y. Honda, O. Kitao, H. Nakai, T. Vreven, J. A. Montgomery Jr., J. E. Peralta, F. Ogliaro, M. J. Bearpark, J. Heyd, E. N. Brothers, K. N. Kudin, V. N. Staroverov, R. Kobayashi, J. Normand, K. Raghavachari, A. P. Rendell, J. C. Burant, S. S. Iyengar, J. Tomasi, M. Cossi, N. Rega, N. J. Millam, M. Klene, J. E. Knox, J. B. Cross, V. Bakken, C. Adamo, J.

Jaramillo, R. Gomperts, R. E. Stratmann, O. Yazyev, A. J. Austin, R. Cammi, C. Pomelli, J. W. Ochterski, R. L. Martin, K. Morokuma, V. G. Zakrzewski, G. A. Voth, P. Salvador, J. J. Dannenberg, S. Dapprich, A. D. Daniels, Ö. Farkas, J. B. Foresman, J. V. Ortiz, J. Cioslowski and D. J. Fox, *Gaussian 09*, 2009.

6. R. J. Mandle and J. W. Goodby, *Phys Chem Chem Phys*, 2019, DOI: 10.1039/C9CP00736A.
